# DSGA-Net: Deeply separable gated transformer and attention strategy for medical image segmentation network

**DOI:** 10.1016/j.jksuci.2023.04.006

**Published:** 2023-04-19

**Authors:** Junding Sun, Jiuqiang Zhao, Xiaosheng Wu, Chaosheng Tang, Shuihua Wang, Yudong Zhang

**Affiliations:** a School of Computer Science and Technology, Henan Polytechnic University, Jiaozuo, Henan 454000, PR China; b School of Computing and Mathematical Sciences, University of Leicester, Leicester LE1 7RH, UK; c Department of Information Systems, Faculty of Computing and Information Technology, King Abdulaziz University, Jeddah 21589, Saudi Arabia

**Keywords:** Medical image segmentation, Transformer, Gated attention mechanism, Depth separable

## Abstract

To address the problems of under-segmentation and over-segmentation of small organs in medical image segmentation. We present a novel medical image segmentation network model with Depth Separable Gating Transformer and a Three-branch Attention module (DSGA-Net). Firstly, the model adds a Depth Separable Gated Visual Transformer (DSG-ViT) module into its Encoder to enhance (i) the contextual links among global, local, and channels and (ii) the sensitivity to location information. Secondly, a Mixed Three-branch Attention (MTA) module is proposed to increase the number of features in the up-sampling process. Meanwhile, the loss of feature information is reduced when restoring the feature image to the original image size. By validating Synapse, BraTs2020, and ACDC public datasets, the Dice Similarity Coefficient (DSC) of the results of DSGA-Net reached 81.24%,85.82%, and 91.34%, respectively. Moreover, the Hausdorff Score (HD) decreased to 20.91% and 5.27% on the Synapse and BraTs2020. There are 10.78% and 0.69% decreases compared to the Baseline TransUNet. The experimental results indicate that DSGA-Net achieves better segmentation than most advanced methods.

## 1 Introduction

Medical image segmentation accurately describes organs, lesions, and other regions in medical images. It can help doctors make accurate and rapid clinical diagnoses and pathological research analyses. In clinical diagnosis, organ labeling requires personnel with solid professional knowledge to do it manually, which is time-consuming, laborious, and tedious. There are many problems to solve in medical image segmentation tasks, such as insufficient data and inaccurate labeling positions. Therefore, it has always been the focus of research in this field to realize medical image segmentation using computational vision (Yao et al., 2020; Liu et al., 2021; Cheng et al., 2022).

In recent years, with the development of deep learning technology, convolutional neural networks (CNN) have been widely used in medical image segmentation (Mu and Li, 2019; Philbrick et al., 2019; Tian et al., 2020). Long *et al*. (Long et al., 2015) proposed the Full Convolutional Network (FCN), which replaces the full connection layer with the convolution layer to extract image features and uses the upsampling layer to restore the image to its original size to obtain a more refined segmentation effect. Ronneberger *et al*. (Ronneberger et al., 2015) advance the U-Net network. It is based on the symmetric structure of the En-Decoder. It uses the skip connection to realize the transmission of the characteristic information between the En-Decoder, thus obtaining better segmentation performance. Zhang *et al*. (Diakogiannis et al., 2020) proposed a Residual Connection UNet (ResUNet), which replaces each submodule of U-Net with a residual connection module to obtain the deeper characteristics of the network, alleviate the problem of gradient disappearance and increase the convergence speed of the network. Oktay *et al*. (Oktay et al., 2018) proposed the Attention to U-Net (Atten-UNet) to enhance the sensitivity of the model to feature information. Zhan *et al*. (Zhan et al., 2023) proposed the Multi-view Attention Mechanism and Adaptive Fusion Strategy (CFNet) network. It uses Multi-view Attention Mechanism (MAM) for feature extraction and cross-scale feature fusion in skip connection to effectively extract feature information in multiple receptive fields. The Fusion Weight Adaptive Allocation Strategy (FAS) is adopted to effectively guide the cross-scale fusion feature information input to the decoder to solve the semantic difference problem. Although CNN-based networks achieve good segmentation results, due to the innate boundedness of convolution and the difficulty in interacting with remote semantic information. It is impossible to model the feature information of remote dependencies in the feature graph, resulting in insufficient context feature information capture (Gu et al., 2018; Sun et al., 2019; Zhou, 2020).

To make up for the insufficiency of convolution, scholars introduced the Vision Transformer (Dosovitskiy et al., 2020) (ViT) model in the field of Natural Language Processing (NLP) into the field of medical image segmentation. The self-attention mechanism in the Transformer can solve the problem of remote information interaction, thus obtaining global feature information (Bitter et al., 2010; Khurana et al., 2023; Rezaii et al., 2022). Chen *et al*. (Chen et al., 2021) proposed the Transformers Make Strong Encoders (TansUNet) network, which combines Transformer with CNN to catch global spatial feature information and local feature information at the same time. However, when CNN upsamples the feature map in the Decoder part, the limitation that the convolved receptive field is too small in restoring the global feature to the initial resolution remains unchanged, which will disturb the segmentation of small organs in medical images. Hei *et al*. (Heidari et al., 2023) proposed the Hierarchical Multi-scale Representations Using the Transformers (HiFormer) network, which combines CNN and Transformer to obtain global and local features and designs a Double-Level Fusion (DLF) module on the hop connection. However, only global feature information can be obtained in the skip connection. It is difficult to obtain local feature information in the skip connection. Yuan *et al*. (Yuan et al., 2023) proposed CNN and Transformer Comprehensive Network (CTC-Net) based on U-Net. The encoder uses a combination of CNN and Swin-Transformer to propose a Cross-domain Fusion Block (CFB) to fuse the feature information extracted by CNN and Swin Transformer. A Feature Complementary Module (FCM) is proposed to improve feature representation, which combines cross-domain fusion, feature correlation, and dual attention. Gao *et al*. (Cao et al., 2022) proposed the Unet-like Pure Transformer (SwinUNet) network, which adopts the pure Swin Transformer method in the En-Decoder. However, Swin Transformer focuses on the interaction of global feature information. It neglects the extraction of low-resolution features, resulting in the introduction of a large amount of irrelevant information in the fusion of low-resolution and high-resolution. Li *et al*. (Li et al., 2022) proposed the Separable Vision Transformer (SepViT) network by combining Depth Separable Self-Attention with grouped self-attention. The feature information inside windows and between windows is captured. However, during the upsampling process, many details cannot be recovered, and it isn’t easy to obtain the location information of context features. Gao *et al*. (Gao et al., 2021) proposed an efficient self-attention mechanism and relative position-coding to polymerize global context information at multiple scales. It can be problem-solving that the medical image processing data set is small and insensitive to location information. However, the resolution of shallow feature information is higher, and more location and detail information is not used effectively. Zhang *et al*. (Zhang et al., 2021) proposed the Fusing Transformers and CNNs (TransFuse) network architecture, the new fusion technology module is used to integrate the multi-level characteristics of each branch. However, the shallow information of the Encoder is not effectively used during the upsampling process, and the loss of the feature information recovered from the upsampling is not alleviated. Chen *et al*. (Chen et al., 2021) proposed the Multi-level Attention-guided U-net with Transformer (TransAttUnet) network, designed multilevel lead attention and multiscale skip connection, which can mitigate the loss of fine details caused by convolution stack and continuous sampling operation. However, the feature information of the same layer with low resolution cannot be obtained at each level of the upsampling.

To solve the above problems, we make the following contributions:

(1) We propose the Deep Separable Gate Visual Transformer (DSG-ViT) Block. It can realize local information interaction within windows, global information between windows, and global information interaction between channels. While extracting features, the Deep Separable Gated Attention mechanism is used to increase the sensitivity of location information, which can solve the feature selection of organ location information and reduce the possibility of the organ being wrongly segmented.
(2) We propose the Mixed Three Branch Attention (MTA) module, which will extract the rich semantic information of each Encoder layer and the accurate target location information to fuse with the Decoder and then compensate for the detailed information lost in the downsampling process and increase the feature information in the upsampling process.
(3) We propose a novel medical image segmentation network called DSGA-Net, which uses a 4-layer Depth Separable Gated Visual Transformer (DSG-ViT) module as the Encoder part and a Mixed Three-branch Attention (MTA) module for feature fusion between each layer of the En-Decoder to obtain the final segmentation results, which achieves better results than previous models.

The rest of the paper is summarized as follows: Section 2 introduces the related work of the paper, Section 3 introduces the principles of the methods used and the overall structure of the model, Section 4 introduces the experimental dataset and results required for the experiment, and Section 5 is the conclusion.

## 2 Related work

### 2.1 Medical image segmentation based on CNN

The development of medical image segmentation is a process from manual segmentation to automatic computer segmentation. Currently, most medical treatments use computers to complete image segmentation alone, which has also become a hot issue for scholars at home and abroad to study.

With the wide application of CNN, FCN has achieved excellent results in medical image segmentation. Long *et al*. (Long et al., 2015) proposed the Full Convolution Network (FCN), which replaces the full connection layer with the convolution layer to extract the image features and uses the upper sampling layer to restore the image to its original size to obtain the segmentation results. The segmentation results of FCN, which is stacked in order, are usually very rough, so scholars have improved the FCN model. U-Net is an FCN model of Encoding and Decoding structure. It uses U-type Encoding and Decoding structures to obtain more feature information and improve segmentation accuracy.

In the CNN-based method, multi-scale fusion has been proven to improve the segmentation performance further. Based on the U-Net structure that has been introduced, Redesigning Skip Connections to Exploit Multiscale Features (U-Net++) (Zhou et al., 2019) and A Full-scale Connected Unet (U-Net 3++) (Huang et al., 2020) are proposed to add multi-scale structure on the jump connection between the Encoder and Decoder to extract richer context information and reduce the semantic difference between the low Encoder and the deep Decoder. Zhan *et al*. (Zhan et al., 2023) proposed the Multi-view Attention Mechanism and Adaptive Fusion Strategy (CFNet) network, which uses the novel Multi-view Attention Mechanism (MAM) for feature extraction and cross-scale method for feature fusion to obtain more accurate cross-scale fusion features in jump connection. At the same time, the Fusion Weight Adaptive Allocation Strategy (FAS) is adopted to effectively input the cross-scale fusion features to the Decoder to solve the semantic gap problem.

### 2.2 Medical image segmentation based on transformer

Due to the limited receptive field of the CNN model, the long-term dependence on modeling is limited. In order to make up for the limitations of CNN, Transformer’s model has become more and more widely used in medical image segmentation.

Cao *et al*. (Cao et al., 2022) proposed the SwinUnet model based on a pure Swin-Transformer’s U-shaped structure for segmenting two-dimensional medical images. In addition to applying pure Transformer, Chen *et al*. (Chen et al., 2021) combined CNN and Transformer to propose the TransUnet structure, by which CNN captures local feature information. Transformer captures global feature information, and the two combine to compensate for each other’s shortcomings. Gao *et al*. (Gao et al., 2021) proposed a UNEt TRansformers (UNETR) network, using a Transformer-based encoder for feature extraction and CNN based Decoder for final 3D medical image segmentation. In the past, the Encoder combined CNN and Transformer to extract the local and global information, often ignoring the important feature information between channels. The channel and spatial attention based on CNN is used in the skip connection between codecs. Because of the limitations of CNN, some important feature information will be lost.

We advance a novel medical image segmentation network model to solve the above problem with a Depth Separable Gating Transformer and a Three-branch Attention module (DSGA-Net). The model adds a Depth Separatable Gated Visual Transformer (DSG-ViT) module to its Encoder to extract features from global, local, and inter-channel feature information. Secondly, a Mixed Three-branch Attention (MTA) module is proposed to increase the number of features in the upsampling process. At the same time, when the feature image is restored to the original image size, the loss of feature information is reduced, and the accurate segmentation structure is achieved. Through verification on the Synapse, BraTs2020, and ACDC public datasets, the Dice similarity coefficients of DSGA-Net reached 81.24%, 85.82%, and 91.34%, respectively. Moreover, the Hausdorff Score (HD) was reduced to 20.91% and 5.27% on the Synapse and BraTs2020, and there are 10.78%, and 0.69% drops compared to the Baseline TransUNet. Experimental results show that the proposed method achieves better segmentation results compared to similar methods.

### 2.3 Separable visual transformer (SepViT)

Li *et al*. (Li et al., 2022) advance an efficient Transformer back-bone called Separable Visual Transformer (SepViT). Its key design is Separable Self-Attention (Sep-Attention), which is made up of Deepwise Self-Attention (DWA) (Li et al., 2022) and Pointwise Self-Attention (PWA) (Li et al., 2022).

DWA is used to capture the local features inside each window. Each window can be regarded as an input channel of the characteristic diagram. Different windows cover different information and create a win token for each window, which can integrate the spatial information in each channel.

(1) $$\text{DWA}(z)=\text{Attention}(z\cdot W_{Q},z\cdot W_{K},z\cdot W_{V})$$

where *z* is the feature tokens, which consist of the pixel and window tokens. *W_Q_*, *W_K_*, and *W_V_*, denote three linear layers for query, key, and value computation in a regular self-attention. Attention means a standard self-attention operator that works on local windows.

PWA establishes the relationship between windows through window markers, mainly used to fuse cross-window information. The feature map and window mark are extracted from the output of DWA, and the attention map is finally generated through the Normalization Layer (LN) and a Gaussian Error Linear Unit (GELU) (Li et al., 2022) activation function. At the same time, the feature graph is used as a branch of value in PWA, and the attention graph is used to calculate the attention between windows, thus realizing the global information interaction.

(2) $$\text{PWA}(z,\omega t)=\text{Attention}\{\text{GELU}[\text{LN}(\omega t)]\cdot W_{Q},\text{GELU}[\text{LN}(\omega t)]\cdot W_{K},z\text{\}}$$

where *ωt* denotes the window token. Attention is a standard self-attention operator but works on all of the windows *z*.

### 2.4 Attention mechanism

**Fig. 1:**
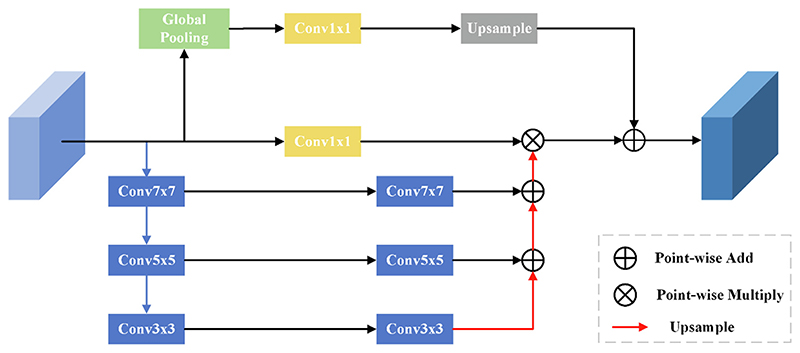
Spatial feature pyramid attention mechanism module.

Li *et al*. (Li et al., 2018) proposed the Pyramid Attention Network (PAN) network model. Its key design is the spatial feature pyramid attention module and the global attention upsampling module. The inter-feature pyramid attention module mainly uses different convolution kernels to draw feature information of different scales and then fuses the extracted feature information of different scales. It is helpful to obtain the correlation of adjacent feature information more accurately. Meanwhile, the similarity between features is extracted by multiplying the attention map after high-level semantic information and multi-scale fusion. Finally, the output results are added to the global attention upsampling. Its basic structure is shown in Fig. 1.

The global attention upsampling module comprises the global average pooling layer, 1 × 1 the convolution layer, and the upsampling layer. It uses the global average pooling to obtain the global semantic information.

## 3 Methodology

### 3.1 Proposed deep separable gate visual transformer (DSG-ViT)

**Fig. 2:**
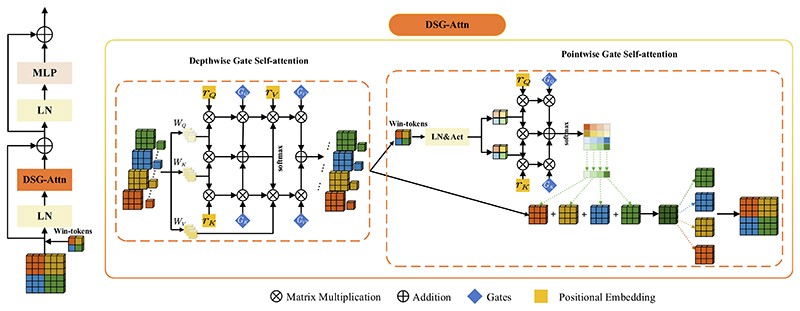
DSG-ViT.

Although the SepViT (Li et al., 2022) can learn global feature information within and between windows well by window tokens, SepViT is not accurate in learning the location of organs when training medical image segmentation data. Gao *et al*. (Gao et al., 2021) believed that Transformer needs a great quantity of data for training if it wants to learn accurate position deviation, and for the experiment of medical image segmentation with a small-scale data set, the position information learned has a large error. Shaw *et al*. (Shaw et al., 2018) have proved that relative position-coding can encode the spatial structure of an image, so it is not always accurate in remote interaction. We propose a Deep Separable Gated Visual Transformer module (DSG-ViT) to solve this problem, as shown in Fig. 2.

The left side is the overall framework of DSG-ViT. We input tokens with window marks into the Deep Separable Gated Self-Attention (DSG-Attn) mechanism and then input the results of DSG-Attn into a layer of LN and MLP operations, fusing features of different self-attention forces.

In addition, we use residual structures to mitigate the phenomenon of gradient explosion and disappearance. The right side of Fig. 2 shows the internal structure of DSG-Attn. DSG-Attn consists of Depthwise Gate Self-Attention (DWGA) and Pointwise Gate Self-Attention (PWGA). Its basic principle is that based on Sep-Attn, it introduces the relative position-coding with a gated self-attention mechanism, embeds the position-coding into the DWA and the PWA, and introduces the gating mechanism that can control the position deviation to the global upper and lower coding, forming DWGA and PWGA. The Deeply Separable Gated Self-Attention mechanism (DWGA) is defined as follows:

(3) $$D_{o}=z\sum_{p\in N_{m\times m}(o)}\text{softma}{\text{x}}_{p}(q_{o}^{T}k_{p}+G_{Q}q_{o}^{T}r_{p-o}^{q}+G_{K}k_{p}^{T}r_{p-o}^{k})(G_{V1}v_{p}+G_{V2}r_{p-o}^{v})$$

Given an input feature map *X* ∈ ℝ^*C×H×W*^, *C* denotes the channel number, *H* denotes the height of the feature map, *W* denotes the width of the feature map, *z* denotes the feature tokens, learning the global features of each window. *N* denotes the pixel of the whole feature map, *o* denotes a pixel (*i, j*) in the feature map, *N_m×m_*(*o*) is the local area of *m × m* size centered on position:

(4) $$\left\{ \begin{array}{l} o=(i,j)\\ q_{o}=W_{Q}x_{o}\\ k_{o}=W_{K}x_{o}\\ v_{o}=W_{V}x_{o} \end{array}\right.$$

which is a linear mapping of *x*_*o*_∀*o* ∈ *N*, and *W_Q_*, *W_K_* and *W_V_* are learnable parameters. Since $q_{o}^{T},$ *k_p_* and *v_p_* do not contain any position information, the relative position deviation terms $r_{p-o}^{q},r_{p-o}^{k}$ and $r_{p-o}^{\nu }$ are added for $q_{o}^{T},$ *k_p_* and *v_p_*, respectively. $q_{o}^{T}r_{p-o}$ indicates the correlation from position *p* = (*a, b*) to position *o* = (*i, j*), *G_Q_*, *G_K_*, *G*_*V*1_ and *G*_*V*2_ are the four learnable gated position embeddings. It provides information on whether spatial location can be learned correctly for the positional bias term and controls the effect of the learned relative position encoding on the accuracy of encoding global context information.

If a relative position code is accurately learned, the gating mechanism will give it a higher weight than those codes without accurate learning position information. In the case of the global receptive field, it can obtain more accurate spatial location information.

Li *et al*. (Li et al., 2022) proposed window markers and used PWA with window markers to fuse cross-window feature information, to better capture the feature information between windows. Position coding and controllable position deviation gating mechanisms are introduced to capture the feature information and accurately obtain the spatial position information between windows. Extract feature map and window tokens (*ωt*) from the output of DWGA, use window tokens and gating mechanism with position coding to build the attention relationship between windows and pose attention map. At the same time, the feature map is directly taken as the value matrix, and the attention calculation is carried out between windows using the attention map and the feature map to realize the global information interaction.

PWGA is calculated as follows:

(5) $$P_{o}=z\cdot \omega t\sum_{p\in N_{m\times m}(o)}\text{softma}{\text{x}}_{p}(q_{o}^{T}k_{p}+G_{Q}q_{o}^{T}r_{p-o}^{q}+G_{K}k_{p}^{T}r_{p-o}^{k})$$

where *z* is the feature token, *ωt* denotes the window token, *q_o_* = *W_Q_x_o_*, *k_o_ = W_K_x_o_* and *v_o_ = W_V_x_o_* is a linear mapping of *x_o_*∀*o* ∈ *N*.

### 3.2 Proposed mixed three-branch attention mechanism

**Fig. 3:**
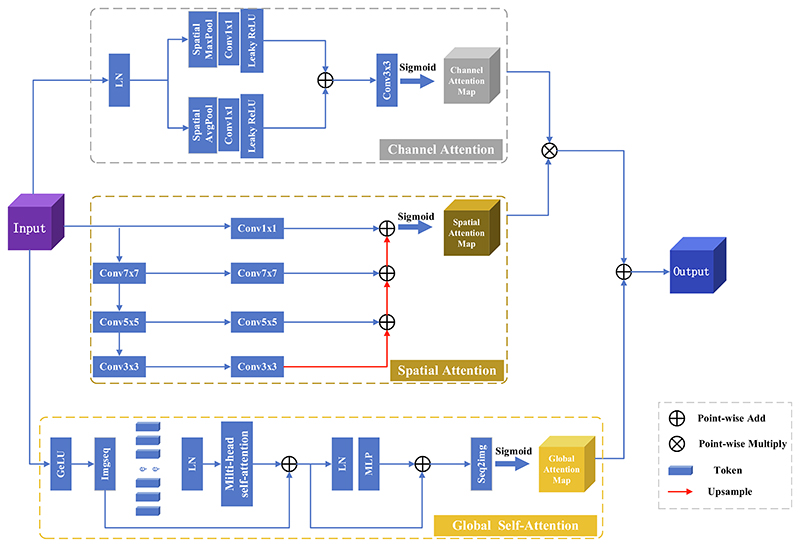
MTA Block.

Mixed Three-branch Attention (MTA) is a mixed attention model which combines channel attention, spatial attention, and global context self-attention. It can map features from the three dimensions of channel, space, and global context, comprehensively improve the loss of extracted feature information and provide accurate feature information for the Decoder in recovering the feature map to the original size. We input the extracted feature map of each DSG layer into the corresponding MTA module for feature extraction and then perform feature fusion with the decoder of the corresponding layer. The structure is shown in Fig. 3.

Channels focus on the feature relationship between channels, mainly capture image texture features, and obtain information about each channel through maximum and average pooling, respectively. Given an input feature map *X* ∈ ℝ^*C×H×W*^, denotes the number of channels, *H* denotes the height of the feature map, *W* denotes the width of the feature map. MaxPooling and AveragePooling are used to extract feature information along the spatial axis, then feature maps *C_M_* ∈ ℝ^*C*×1×1^ and *C_A_* ∈ ℝ^*C*×1×1^ are respectively 1 × 1 convolved, LeakyReLU activated, and then perform element-based addition operations. Finally, they use Sigmoid functions to obtain channel attention maps. The specific process is as follows:

(6) $$C_{M}=\;\text{LeakyReLU\{}f_{\mathrm{Conv}\;}[\text{MaxPool}(X)]\text{\}},C_{M}\in {\mathbb{R}}^{C\times 1\times 1}$$

(7) $$C_{A}=\;\text{LeakyReLU\{}\;f_{\mathrm{Conv}\;}[\text{AveragePool}\;(X)]\text{\}},C_{M}\in {\mathbb{R}}^{C\times 1\times 1}$$

(8) $$C(X)=\text{Sigmoid}(C_{M}⊕C_{A}),C(X)\in {\mathbb{R}}^{C\times 1\times 1}$$

where *f_Conv_* denotes the convolution operation. *C_M_* denotes the feature map of the MaxPooling output, *C_A_* denotes the feature map of the AveragePooling output, *C*(*X*) denotes the channel attention map, ⊕ denotes summation by pixel.

Since there is a large semantic difference between deep and shallow information in the downsampling process, and it is challenging to obtain spatial location information at different scales in the feature map, we designed multi-scale spatial attention. The input feature map is extracted with different scale feature information through three convolution kernels of different sizes to form a multi-scale pyramid structure. The convolution kernels are 7 × 7, 5 × 5, and 3 × 3, respectively. The pyramid can effectively fuse spatial information at different scales so that smaller details in the feature map can be effectively segmented in a larger receptive field, and larger features can be effectively segmented in a smaller receptive field.

Given a feature map *X* ∈ ℝ^*C×H×W*^, the features are extracted along the channel axis, the feature vectors at different scales are generated by convolution at three different scales, and the Batch Normalization (BN) and Rectified Linear Units (ReLU) activation function operations are performed after each convolution at different scales. The specific operations are as follows:

(9) $$S_{p}=\text{ReLU}\{\text{BN}[f_{1,p}^{k\times k}\{\text{ReLU}[\text{BN}(f_{2,p}^{k\times k}(X))]\}]\}$$

where *S_p_* denotes the feature vector, $f_{2,p}^{k\times k}$ denotes the convolution operation with a convolution kernel of *k*, the stride of 2, and the padding of *p*, with a convolution kernel size of *k* = {3, 5, 7}, and a corresponding padding size of *p* = {1, 2, 3}. The output of the multi-scale feature pyramid is the sum of the weights at different scales *T*:

(10) $$T=\sum_{p=1}^{3}S_{p},T\in {\mathbb{R}}^{1\times H\times W}$$

Finally, use the Sigmoid function to obtain the spatial attention map *S*:

(11) $$S=\text{Sigmoid}(T),S\in {\mathbb{R}}^{1\times H\times W}$$

Global self-attention uses the Vision Transformer (Dosovitskiy et al., 2020) (ViT) network structure. ViT performs global modeling when acquiring feature information. The Multi headed Self-attention mechanisms enable the construction of correlations of global features that can capture global feature information. It can compensate for information loss in spatial and channel attention.

First, the feature map *X* ∈ ℝ^*C×H×W*^ is divided into patch sequences *X_P_* ∈ ℝ^*C×P×P*^, which (*P*, *P*) denotes the size of each patch. Since the patch sequence has no location information, it is necessary to add a learnable location code *c_loc_* ∈ ℝ^*C×P×P*^ to the patch sequence and then obtain the attention sequence through a layer of LayerNormalization (LN). The attention sequence is input to the Multi-Headed Self-Attention (MHSA) to compute the similarity feature representation between patches. Then, a layer of LN and MLP operations are performed to fuse the feature extraction from different self-attentions. In addition, a residual structure is used to mitigate the occurrence of gradient explosion and vanishing phenomena. Finally, the feature sequence is transformed into a global attention map *G* ∈ ℝ^*C×H×W*^. The concrete implementation can be expressed as follows:

(12) $$\begin{array}{c} g_{mhsa}=\text{MSHA}\{\text{LN}[f_{\mathrm{flat}\;}(X)⊕c_{\mathrm{loc}\;}]\}⊕\{\text{LN}[f_{\mathrm{flat}\;}(X)]\},g_{mhsa}\in {\mathbb{R}}^{C\times P\times P} \end{array}$$

(13) $$g_{mhsa}^{\prime }=\text{MLP}[\text{LN}(g_{mhsa})]⊕g_{mhsa},g_{\mathrm{mhsa}\;}^{\prime }\in {\mathbb{R}}^{C\times P\times P}$$

(14) $$G=\text{SeqImg}(g_{mhsa}^{\prime }),G\in {\mathbb{R}}^{C\times H\times W}$$

where *f_flat_* denotes a flatten operation, SeqImg denotes the operation of converting a sequence of features into a global attention map, *G* ∈ ℝ^*C×H×W*^ denotes a global attention map, ⊕ denotes a pixel-by-pixel summation.

Multiply the obtained channel attention map *C* ∈ ℝ^*C*×1×1^ with the spatial attention map *S* ∈ ℝ^1×*H*×*W*^ to obtain the channel-space attention map *CS* ∈ ℝ^*C×H×W*^ and finally, add *CS* with the global attention map *G* to obtain the mixed three-branch attention map MTA ∈ ℝ^*C×H×W*^.

(15) $$\text{MTA}=(C⊗S)⊕G,\;\text{MTA}\;\in {\mathbb{R}}^{C\times H\times W}$$

where MTA ∈ ℝ^*C×H×W*^ denotes a mixed three-branch attention graph, ⊗ denotes multiplication using the broadcast mechanism in Python, and ⊕ denotes summation by elements.

### 3.3 Network structure of proposed DSGA-Net

**Fig. 4:**
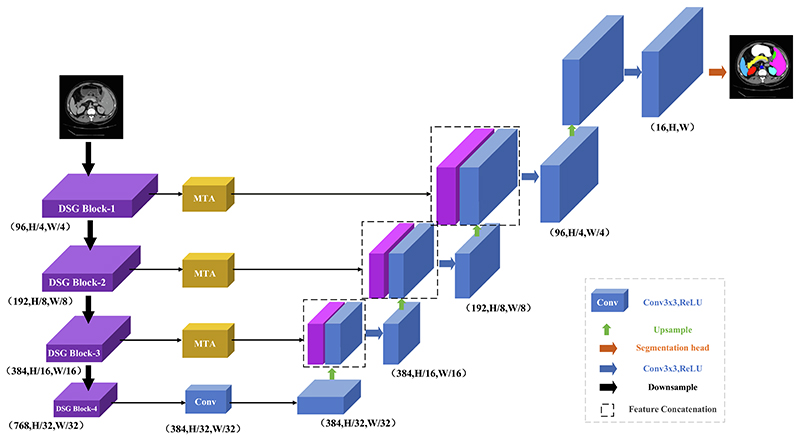
Structure of the proposed DSGA-Net.

In response to the problem of insufficient feature location information extracted by the currently available models and the loss of feature information in the upsampling process, we advance the DSGA-Net method. The overall structure of the proposed DSGA-Net consists of three parts: (i) The 4-layer Deep Separable Gate (DSG) Block, which constitutes the Encoder, (ii) the cascaded upsampling Decoder module, and (iii) the Mixed Three-branch Attention (MTA) module between the En-Decoder. The network structure is shown in Fig. 4.

**Fig. 5:**
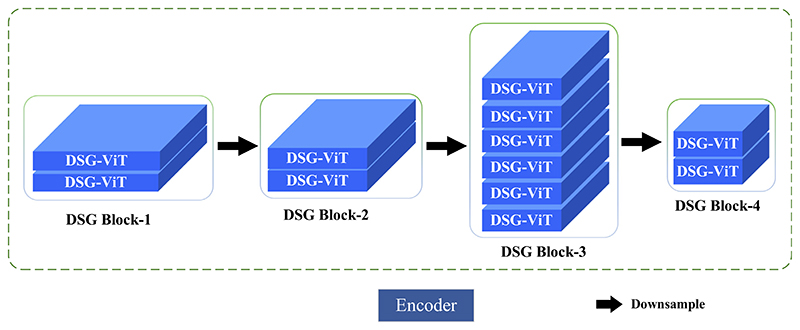
Structure of the Encoder.

The DSG Block that constitutes the Encoder has four layers, as shown in Fig. 5: DSG Block-1, DSG Block-2, DSG Block-3, and DSG Block-4. Each layer is composed of a different number of DSG-ViTs. The numbers of DSG-ViTs in each layer are 2, 2, 6, and 2, respectively. Each layer has a patch merge layer to downsample the preprocessed image and then input it to the DSG Block in the next layer to acquire the feature form of the feature map to extract the detailed feature information of organs effectively.

Because the shallow feature map is high resolution and contains more detailed features, the output of each layer of DSG Block is sent to the skip connection MTA to capture the feature details at different angles to generate the output of the Decoder. It uses low-level feature information to increase the receptive field at the upsampling stage and irrecoverable details. In the last layer of Encoder, because the underlying feature map is too small but contains rich feature information, only 3 × 3 convolution is used to transform the number of channels instead of using the MTA module.

Each layer in the Decoder consists of a 2 × 2 upsampling operator, 3 × 3 convolution, and Relu activation function. Finally, the number of channels in the feature map reconstructed by 1 × 1 convolution is reduced to the number of classes, and the final segmentation result is predicted.

## 4 Experiments, results, and analysis

### 4.1 Experimental dataset

**Synapse Multi-Organ Segmentation Dataset** (https://www.synapse.org/#!Synapse:syn3193805/wiki/217789, n.d.): We used the 30 abdominal CT scanning images and obtained 3779 axial enhanced abdominal clinical CT images. Each abdominal CT was composed of 85 ~ 98 slices with a slice pixel 512 × 512. We randomly divided the data according to a certain proportion, including 18 cases (2212 axial slices total). We trained eight abdominal organs (aorta, gallbladder, left kidney, right kidney, liver, pancreas, spleen, stomach), and 12 cases were verified. Among them, the slices of 18 training and 12 validation data are independent and do not overlap.

**Automated Cardiac Diagnostic Challenge Dataset** (https://www.creatis.insa-lyon.fr/Challenge/acdc/, n.d.): The ACDC dataset is an electronic MRI image collected while examining different patients with an MRI scanner. Each patient’s MRI image contains 18 ~ 20 slices while all being manually labeled by the physician with the true values of the left ventricle (LV), right ventricle (RV), and myocardium (MYO). We randomly divided it into 70 training samples (1930 axial slices), ten validation samples, and 20 test samples. Similar to (Chen et al., 2021), only the mean DSC was used to evaluate our model on this dataset.

**Brain Tumor Segmentation Challenge 2020 Dataset** (https://www.kaggle.com/datasets/awsaf49/brats20-dataset-training-validation, n.d.): The BraTs2020 dataset has 369 brain tumor cases for model training and 125 cases for model validation. Each case contains four MRI scans of different modes (Flair, T1, T1ce, and T2), which are marked by experts in the field. Labels are divided into four categories: background, NCR/NET, ED, and ET. The evaluation results are based on three different brain tumor regions: the Whole Tumor (WT) region, the Tumor Core (TC) region,

(16) $$\left\{ \begin{array}{l} \text{WT}=\frac{\text{NCR}}{\text{NET}}+\text{ED}+\text{ET}\\ \text{TC}=\frac{\text{NCR}}{\text{NET}}+\text{ET} \end{array}\right.$$

and the Enhanced Tumor region (ET).

### 4.2 Experiment setup and evaluation indicators

We use a 64-bit Windows 10 system with Nvidia GeForce RTX 2070 s graphics card based on Python 3.8 and Python 1.10.1 frame-works. During the training, simple data enhancement operations using flips and rotations were performed for all training cases.

We set the input feature map size to 224 × 224. The model is trained with the SGD optimizer. The learning rate is set to 0.01. The momentum parameter is set to 0.9. The weight recession is set to 0.0004, and the Batch size is set to 24. The evaluation metrics are used with the average Dice Similarity Coefficient (DSC) and the average Hausdorff Score (HD). DSC is used to measure the similarity of two regions, and the value is in the range of [0,1]. The larger the value, the more similar the two regions are. HD is used to calculate the distance between two regions. The smaller the value, the higher the similarity between the two regions.

### 4.3 Ablation experiments

We conducted ablation experiments under the Synapse multi-organ segmentation dataset to investigate the effect of different factors on the model. Specifically: (1) DSG-ViT with MTA ablation experiments; (2) the effect of the number of skip connections; (3) The effect of input resolution; (4) The effect of the number of DSG-ViT modules.

#### 4.3.1 Influence of the DSG-ViT and MTA

To verify the effectiveness of DSG-ViT and MTA modules in DSGA-Net network architecture, we conducted four sets of experiments separately using TransUNet (TU) (Chen et al., 2021) as Base-line. As shown in Table 1, the first group is Baseline, the second group adds the DSG-ViT module to Baseline, the third group adds the MTA module to Baseline, and the fourth group adds both DSG-ViT and MTA modules to Baseline.

The experimental results are shown in Table 2. Compared with the first Baseline model, the second TU+(DSG-ViT) model improved the evaluation index DSC by 1.06% and reduced HD to 23.34%. The evaluation index DSC of the third TU + MTA model improved by 1.27%, and HD decreased to 23.65%. The fourth group is our model DSGA-Net, whose evaluation index DSC improved by 3.76%, and HD decreased to 20.91%.

**Fig. 6:**
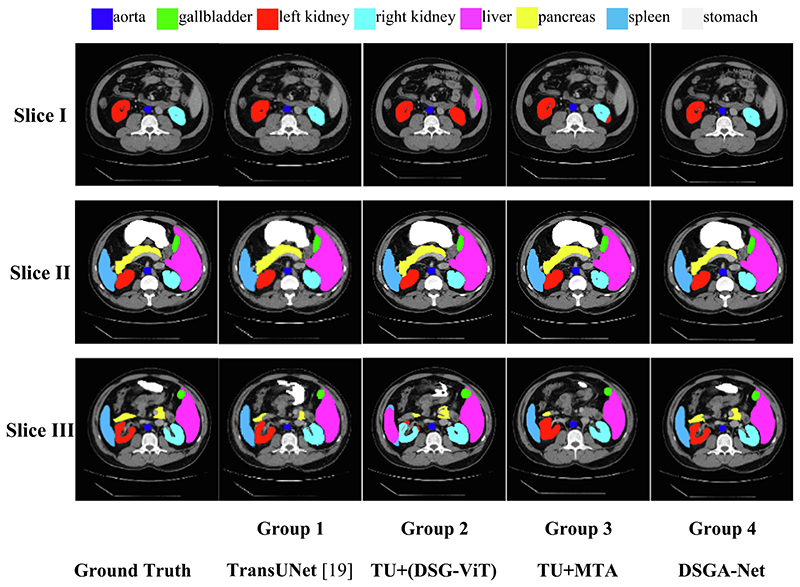
Comparison of DSGA-Net modules segmentation.

Fig. 6 shows an example of visual segmentation for the above effect. We can see from the second group that when the MTA module was removed, there was over-segmentation of organs, such as mis-segmentation of the spleen as the liver, mis-segmentation of the left kidney as the right kidney (Fig. 6, Slice I, Group 2) and over-segmentation of the pancreas (Fig. 6, Slice II, Group 2).

The third group shows that when the DSG-ViT module was removed, the prediction of organ edges was not accurate enough, such as over-segmentation of the internal edges of the left kidney, under-segmentation of the edges of the stomach as well as the liver (Fig. 6, Slice III, Group 3), and rough edge segmentation of the pancreas with insufficient segmentation of the edge protrusions (Fig. 6, Slice II, Group 3).

#### 4.3.2 Influence of the number of skip connections

To verify the influence of the amount of MTAs between En-Decoder on the segmentation effect, we experimentally tested four cases of no MTA (0-MTA), adding MTA (1-MTA) between DSG Block-1 and Decoder only, adding MTA (2-MTA) between DSG Block-1, DSG Block-2, and Decoder respectively, and adding MTA (3-MTA) between DSG Block-1, DSG Block-2, DSG Block-3, and Decoder respectively.

**Fig. 7:**
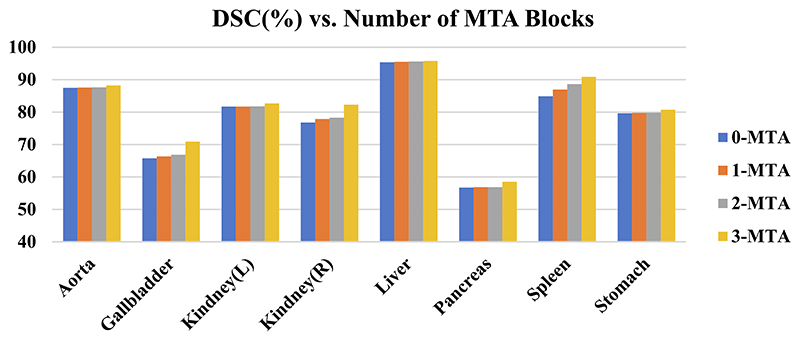
Study of the number of skip connections added to DSGA-Net.

The experimental results are shown in Fig. 7, which shows the performance of the number of skip connections on the segmentation of 8 organs at the average DSC, and it can be seen from the figure that the 3-MTA case brings better segmentation results. The experimental results also show that the segmentation effect on small organs such as the aorta, gallbladder, kidney, and pancreas has more obvious improvement than large organs such as the liver and stomach. The results fully confirm the capability of the MTA module to draw detailed feature information.

#### 4.3.3 Influence of the size of the input resolution

We chose 224 × 224 and 512 × 512 two different resolutions for our experiments, and also compared our method, DSGA-Net, with TransUNet (Chen et al., 2021). The experimental results are shown in Table 3. From the experimental results, we can find out that when we use 512 × 512 as the input when the size of the segmented slices is consistent, the segmentation effect is improved for both our method and TransUNet (Chen et al., 2021).

This indicates that as the image resolution increases, the segmentation of the network structure also increases. It can also be seen from the experimental results that the change in image resolution has a greater effect on the segmentation effect of the Trans-UNet model and a relatively small effect on the DSGA-Net, which also further indicates that the method in our paper has better robustness. Since high-resolution images increase the computational burden of the network due to the increase in the number of slices, the resolution used in our subsequent experiments is the resolution of 224 × 224.

#### 4.3.4 Influence of the number of DSG-ViTs in the DSG block module

We experimentally verify the impact of different numbers of DSG-ViTs in the Encoder’s DSG Block module on the DSGA-Net models’ segmentation performance. Given that the experimental results of (Liu et al., 2022) show that the number of four-layer modules DSG-ViT forming a ratio of 1:1:3:1 will achieve a better segmentation effect, for this reason, we experimentally tested three cases with the number of four-layer DSG-ViT modules (1, 1, 3, 1), (2, 2, 6, 2), and (3, 3, 9, 3), respectively, noted as “Base”, “Middle-1″, and ”Middle-2″, and the experimental results are shown in Table 4. From the experimental results, we can see that with the number of DSG-ViT modules increasing, the segmentation performance has also been significantly improved by 78.16%, 81.24%, and 82.23%, respectively.

It can also be seen from the experimental results that when the number of DSG-ViT modules is increased from “Base” to “Middle-1″, the segmentation performance is improved by 3.08%. When the number of DSG-ViT modules is increased from ”Middle-1″ to “Middle-2″, the segmentation performance is improved by 0.99%. The transition from ”Middle-1″ to “Middle-2″ will not improve performance, but the increased number of modules will cause greater computing consumption. For this purpose, we use ”Middle-1″ as the Encoder, the structure shown in Fig. 3.

### 4.4 Comparison with state-of-the-art methods

#### 4.4.1 Results for the synapse dataset

**Fig. 8:**
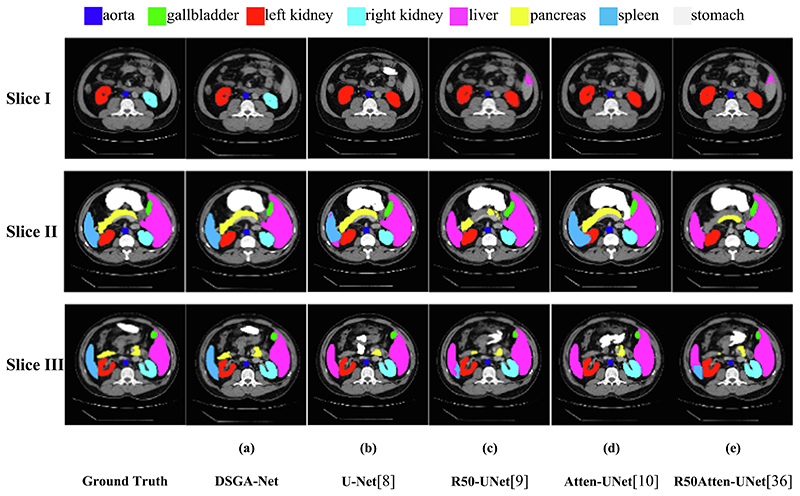
Segmentation results of different CNN-based models on Synapse Dataset.

**Fig. 9:**
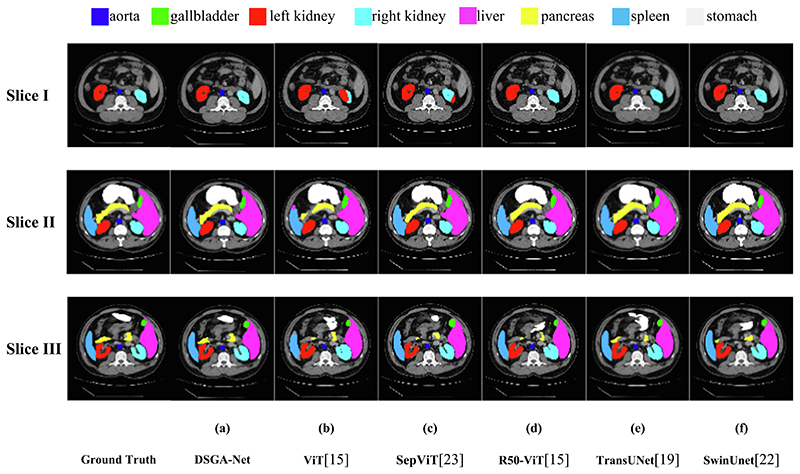
Segmentation Results of Different Variants of CNN and ViT on Synapse Dataset.

To further validate the segmentation performance of our model, we performed two sets of experiments. The first group compares our mode with CNN-based models, including U-Net (Ronneberger et al., 2015), Atten-Unet (Oktay et al., 2018), R50-UNet (Diakogiannis et al., 2020), and R50 Atten-UNet (Schlemper et al., 2019). The second group is the comparison with ViT (Dosovitskiy et al., 2020), CNN, and ViT combined models for comparison, including ViT (Dosovitskiy et al., 2020); SepViT (Li et al., 2022); R50-ViT (Dosovitskiy et al., 2020), TransUnet (Chen et al., 2021); and SwinUnet (Cao et al., 2022). To prove the correctness of the data results, we trained each set of experiments three times and then weighted them to obtain the final experimental results by averaging them. The experimental results are shown in Tables 5 and 6, and Figs. 8 and 9 show the segmentation visualization results of each network structure on the Synapse dataset.

Tables 5 and 6 provide DSC and HD evaluation indicators and computational complexity indicators to reflect the segmentation performance of different methods. Compared to TransUNet and other pure CNN-based frameworks, experimental results show that DSGA-Net pays more attention to boundary information and can achieve better edge prediction.

For pure Transformer methods, DSGA-Net not only ensures sensitivity to boundaries but also prevents feature loss. After adding a deep separable self-attention mechanism and Transformer to traditional CNN, local context information, global context information, and information between channels are fused to improve the expression ability of output features. Then, U shaped structure is used for upsampling recovery to obtain better segmentation results. Our model has the lowest computational complexity using different methods on the Synapse dataset. Still, our model does not significantly reduce computational complexity compared to Trans-UNet, which is also the trend we will study next.

Fig. 8 shows the visualization of segmentation results between our model and other models based only on CNN. Due to the limitations of pure convolution modeling for a long time, the four models are over-segmented, and the segmentation of small organs is not obvious. Specifically, (Fig. 8, Slice I, (b-e)) show that the CNN-only based model identifies the right kidney as the left kidney for segmentation, (Fig. 8, Slice III, (b-e)) shows that the spleen is identified as the liver, and (Fig. 8, Slice II, (b-e)) show that the marginal segmentation of the small organ pancreas is not obvious and appears to be under-segmented.

Fig. 9 shows the segmentation visualization results between our model and the model combined with CNN and Transformer. ViT (Dosovitskiy et al., 2020) and SepViT (Li et al., 2022) are only based on the Transformer model. Because a pure Transformer can exchange global information but has limitations in learning location information; therefore, the two models significantly improved the recognition errors of left and right kidneys in abdominal multi-organ segmentation. However, a small part of the right kidney is still recognized as the left kidney for segmentation (Fig. 9, Slice I, (b-c)), and under-segmentation occurs, such as under-segmentation of the edge details of the spleen and liver (Fig. 9, Slice III, (b-c)). SwinUNet (Cao et al., 2022), TransUNet (Chen et al., 2021), and R50-ViT (Dosovitskiy et al., 2020) are segmentation models based on the combination of CNN and Transformer. It can interact with local and global information, but based on the U-shaped structure, the noise of shallow feature information is not filtered during the jump connection.

These five models make up for the shortcomings of the previous models but have limitations in the accuracy of the segmentation of small organs. For example, the segmentation of small organs such as the pancreas is under-divided, and the segmentation of the stomach is wrong (Fig. 9, Slice III, (d-f)). From (Fig. 9, Slice II, (d-f)), it can be seen that they are not obvious enough to obtain the location information of the pancreatic edge. Thus, the segmentation of the pancreatic edge is too smooth. The experimental results show that the DSGA-Net network structure is more accurate than other segmentation models.

#### 4.4.2 Results for the ACDC dataset

We applied our model’s generalization ability and robustness to the MRI dataset ACDC of automatic heart segmentation for experiments to further verify our model’s generalization ability and robustness. Also, our method is compared with the CNN-based methods R50-UNet (Diakogiannis et al., 2020) and R50 Attend-UNet (Schlemper et al., 2019) and Transformer based methods R50-ViT (Dosovitskiy et al., 2020), SwinUet (Cao et al., 2022) and TransUnet (Chen et al., 2021); respectively, and the experimental results are shown in Table 7. Our model improves the DSC metrics by 3.79%, 4.95%, 3.77%, 1.34%, and 1.63% compared to R50-UNet (Diakogiannis et al., 2020), R50 Atten-UNet (Schlemper et al., 2019), R50-ViT (Dosovitskiy et al., 2020), SwinUnet (Cao et al., 2022) and TransUnet (Chen et al., 2021), respectively. It can be seen that the segmentation performance of our network structure has been significantly improved. In addition, our method has achieved significantly better results in the myocardium (Myo) and left ventricle (LV).

#### 4.4.3 Results for the BraTs2020 dataset

Our model is tested on the brain tumor segmentation data set BraTs2020 to verify the model’s generalization ability again by comparing with the CNN-based methods U-Net (Ronneberger et al., 2015), R50-UNet (Diakogiannis et al., 2020), and R50-Atten-UNet (Oktay et al., 2018) and Transformer based methods SwinUnet (Cao et al., 2022) and TransUnet (Chen et al., 2021), respectively. The experimental results are shown in Table 8. From the experimental results, the segmentation performance based on the Transformer method model is better than that based on the CNN method. Compared with U-Net (Ronneberger et al., 2015), R50-Unet (Diakogiannis et al., 2020), R50-Atten-Unet (Oktay et al., 2018), SwinUnet (Cao et al., 2022), and TransUnet (Chen et al., 2021), the DSC score of our model increased by 5.70%, 5.12%, 3.75%, 0.74%, and 1.52% respectively, and the HD score of our model decreased by 3.07%, 1.78%, 1.73%, 1.22%, and 0.69% respectively. It can be seen that our network structure has good segmentation performance and strong generalization ability. In addition, our method achieves better segmentation results for the whole tumor region (ET) and enhanced tumor region (WT).

## 5 Conclusion

We propose DSGA-Net, a medical image segmentation network with a Deeply Separable Gating Transformer and Attention strategies. Our approach enhances the contextual connection between global and local as well as between channels by adding a Depth Separable Gated Visual Transformer (DSG-ViT) module to the Encoder. We add a Mixed Three Branch Attention (MTA) module between the Encoder and Decoder to further extract the features of each layer of the Encoder through the spatial, channel, and global attention and input these features into the Decoder, increasing the feature information during the upsampling process. Many tests on medical image segmentation tasks have shown that our method provides a better improvement on the mis-segmentation and under-segmentation of small organs in medical image segmentation. Our method has a better segmentation effect and generalization ability than its counterparts.

However, the issue of a considerable computational burden on computers based on Transformer network structure is yet to be solved. Some devices may not support the global self-attention mechanism, which consumes a large amount of GPU memory and may limit the model’s usability.

In the future, we will go on to develop this algorithm and apply it to other segmentation tasks. We will explore ways to improve segmentation while reducing the number of parameters and increasing the speed of the computer.

## Figures and Tables

**Table 1:** Four groups of experiments.

Group	Description	Short Name
1	Baseline	TU
2	Baseline + DSG-ViT	TU + DSG-VIT
3	Baseline + MTA	TU + MTA
4	Baseline + DSG-VIT + MTA	DSGA-Net

**Table 2:** Validation of different modules of DSGA-Net.

Group	Baseline	DSG-ViT	MTA	Average		Aorta	Gallbladder	Kidney (L)	Kidney (R)	Liver	Pancreas	Spleen	Stomach
				DSC↑	HD↓								
1	✓			77.48	31.69	87.23	63.13	81.87	77.02	94.08	55.86	85.08	75.62
2	✓	✓		78.54	23.34	87.48	65.76	81.67	76.76	95.37	56.74	84.86	79.64
3	✓		✓	78.75	23.65	87.79	68.03	81.34	76.88	94.64	55.21	87.79	78.31
4	✓	✓	✓	**81.24**	**20.91**	**88.21**	**70.87**	**82.67**	**82.31**	**95.76**	**58.49**	**90.87**	**80.74**

**Table 3:** Ablation experiments with input resolution.

Method	Resolution	Average	Aorta	Gallbladder	Kidney(L)	Kidney(R)	Liver	Pancreas	Spleen	Stomach
		DSC								
**TransUNet** (Chen et al., 2021)	**224**	77.48	87.23	63.13	81.87	77.02	94.08	55.86	85.08	75.62
**TransUNet** (Chen et al., 2021)	**512**	84.36	90.68	71.99	86.04	83.71	95.54	73.96	88.80	84.20
**DSGA-Net**	**224**	81.24	88.21	70.87	82.67	82.31	95.76	58.49	90.87	80.74
**DSGA-Net**	**512**	85.28	91.31	72.87	86.31	84.90	96.90	72.78	92.69	84.46

**Table 4:** Ablation experiments for the number of DSG-ViT modules.

DSG-ViT	Average	Aorta	Gallbladder	Kidney(L)	Kidney(R)	Liver	Pancreas	Spleen	Stomach
		DSC								
**Base**	78.16	87.34	65.28	81.37	80.69	94.27	56.64	86.31	79.36
**Middle-1**	81.24	88.21	70.87	82.67	82.31	95.76	58.49	90.87	80.74
**Middle-2**	82.23	88.94	71.31	84.09	83.85	96.22	59.21	91.98	82.21

**Table 5:** Comparison of segmentation performance of different CNN-based network structures on the Synapse dataset.

Method	Average		FLPOs (G)↓	Aorta	Gallbladder	Kidney (L)	Kidney (R)	Liver	Pancreas	Spleen	Stomach
	DSC↑	HD↓									
**U-Net** (Ronneberger et al., 2015)	76.85	39.70	65.37	89.07	69.72	77.77	68.60	93.43	53.98	86.67	75.58
**R50-UNet** (Diakogiannis et al., 2020)	74.68	36.87	62.84	84.18	62.84	79.19	71.29	93.35	48.23	84.41	73.92
**R50-AttenUNet** (Schlemper et al., 2019)	75.57	36.97	56.31	55.92	63.91	79.20	72.71	93.56	49.37	87.19	74.95
**Atten-UNet** (Oktay et al., 2018)	77.77	36.02	58.47	**89.55**	68.88	77.98	71.11	93.57	58.04	87.30	75.75
**DSGA-Net(ours)**	**81.24**	**20.91**	**37.26**	88.21	**70.87**	**82.67**	**82.31**	**95.76**	**58.49**	**90.87**	**80.74**

**Table 6:** Comparison of segmentation performance of different models of CNN and ViT on the Synapse dataset.

Method	Average		FLPOs (G)↓	Aorta	Gallbladder	Kidney (L)	Kidney (R)	Liver	Pancreas	Spleen	Stomach
	DSC↑	HD↓									
**ViT** (Dosovitskiy et al., 2020)	61.50	39.61	50.37	44.38	39.59	67.46	62.94	89.21	43.14	75.45	69.78
**R50-ViT** (Dosovitskiy et al., 2020)	71.29	32.87	54.07	73.73	55.13	75.80	72.20	91.51	45.99	81.99	73.95
**SepViT** (Li et al., 2022)	77.77	30.37	41.19	**88.36**	67.49	80.97	77.36	93.21	53.27	88.31	73.21
**TransUnet** (Chen et al., 2021)	77.48	31.69	38.52	87.23	63.13	81.87	77.02	94.08	55.86	85.08	75.62
**SwinUnet** (Cao et al., 2022)	79.13	21.55	42.68	85.47	66.53	**83.28**	79.61	94.29	54.96	90.66	76.60
DSGA-Net(ours)	**81.24**	**20.91**	**37.26**	88.21	**70.87**	82.67	**82.31**	**95.76**	**58.49**	**90.87**	**80.74**

**Table 7:** Segmentation performance of different methods on the ACDC dataset.

Method	Average	RV	Myo	LV
	DSC↑			
**R50-UNet** (Diakogiannis et al., 2020)	87.55	87.10	80.63	94.92
**R50 Atten-UNet** (Schlemper et al., 2019)	86.75	87.58	79.20	93.47
**R50-ViT** (Dosovitskiy et al., 2020)	87.57	86.07	81.88	94.75
**SwinUnet** (Cao et al., 2022)	90.00	88.55	85.62	95.83
**TransUnet** (Chen et al., 2021)	89.71	**88.86**	84.53	95.73
**DSGA-Net(ours)**	**91.34**	88.78	**88.39**	**96.87**

**Table 8:** Segmentation performance of different methods on the BraTs2020 dataset.

Method	Average		ET	TC	WT
	DSC↑	HD↓			
**U-Net** (Ronneberger et al., 2015)	80.12	8.34	75.34	76.39	88.64
**R50-UNet** (Diakogiannis et al., 2020)	80.70	7.05	75.87	77.34	88.90
**R50-Atten-UNet** (Oktay et al., 2018)	82.07	7.00	76.65	80.21	89.34
**SwinUnet** (Cao et al., 2022)	85.08	6.49	80.05	**86.11**	89.08
**TransUnet** (Chen et al., 2021)	84.30	5.96	79.37	85.21	88.31
**DSGA-Net(ours)**	**85.82**	**5.27**	**81.68**	86.00	**89.78**
